# When the allergy alarm bells toll: The role of Toll-like receptors in allergic diseases and treatment

**DOI:** 10.3389/fmolb.2023.1204025

**Published:** 2023-06-22

**Authors:** Mario Wenger, Sophie Grosse-Kathoefer, Amin Kraiem, Erica Pelamatti, Natalia Nunes, Lisa Pointner, Lorenz Aglas

**Affiliations:** Department of Biosciences and Medical Biology, University of Salzburg, Salzburg, Austria

**Keywords:** Toll-like receptors, TLR, allergy, treatment, microbiome, allergic rhinitis, LPS, allergic sensitization

## Abstract

Toll-like receptors of the human immune system are specialized pathogen detectors able to link innate and adaptive immune responses. TLR ligands include among others bacteria-, mycoplasma- or virus-derived compounds such as lipids, lipo- and glycoproteins and nucleic acids. Not only are genetic variations in TLR-related genes associated with the pathogenesis of allergic diseases, including asthma and allergic rhinitis, their expression also differs between allergic and non-allergic individuals. Due to a complex interplay of genes, environmental factors, and allergen sources the interpretation of TLRs involved in immunoglobulin E-mediated diseases remains challenging. Therefore, it is imperative to dissect the role of TLRs in allergies. In this review, we discuss i) the expression of TLRs in organs and cell types involved in the allergic immune response, ii) their involvement in modulating allergy-associated or -protective immune responses, and iii) how differential activation of TLRs by environmental factors, such as microbial, viral or air pollutant exposure, results in allergy development. However, we focus on iv) allergen sources interacting with TLRs, and v) how targeting TLRs could be employed in novel therapeutic strategies. Understanding the contributions of TLRs to allergy development allow the identification of knowledge gaps, provide guidance for ongoing research efforts, and built the foundation for future exploitation of TLRs in vaccine design.

## Introduction

Pattern recognition receptors (PRRs) represent major sensory molecules initiating defense mechanisms against pathogens and are an important communicator between the innate and adaptive immune system (Li and Wu, 2021). These germline-encoded receptors sense highly conserved pathogen-associated molecular patterns (PAMPs) from different sources like bacteria, virus and fungi. In addition, PRRs recognize so-called damage-associated molecular patterns (DAMPs) released by cell necrosis or damaged tissue but also xenobiotic-associated molecular patterns like toxins or pollutants (Behzadi et al., 2021).

Among the different classes of PRRs, Toll-like receptors (TLRs) represent the most preserved class, recognizing a vast range of molecules derived from pathogenic sources, spanning from viral RNA and DNA to bacterial surface structure motifs. Upon TLR activation, numerous defense mechanisms, including the release of reactive oxygen species, pro-inflammatory cytokines and chemokines, such as interleukin (IL)-6, IL-12 and tumor necrosis factor (TNF)-α, and peptides like β-defensin 2 (Cieślik et al., 2021) are triggered. This results in the recruitment of immune cells, e.g., neutrophils, macrophages and dendritic cells (DCs) (Owen et al., 2020), induction of oxidative stress, and in an overall inflammatory microenvironment necessary for pathogen clearance (West et al., 2011; Horibe et al., 2013; Vidya et al., 2018; Luo et al., 2019). TLR signaling also increases macropinocytosis in DCs as well as phagocytosis in monocytic cells supporting the ingestion of pathogens (Bode et al., 2014; Boye et al., 2016).

**FIGURE 1:**
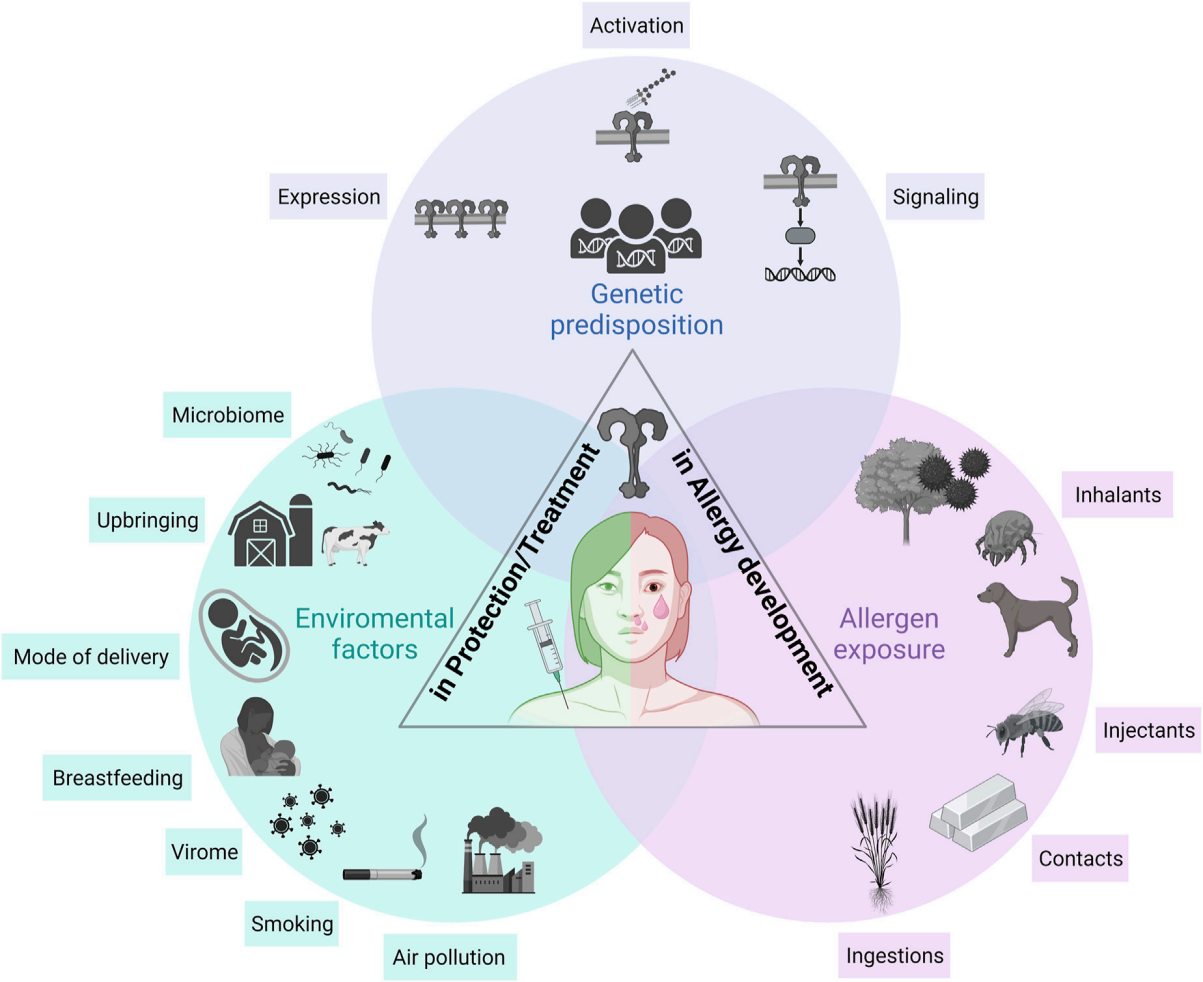
The role of TLRs in allergy development, protection, and treatment. Three major aspects need to be considered when describing the role of TLRs in allergic diseases: (i) the genetic predisposition, (ii) environmental factors influencing TLR responsiveness, and (iii) allergen sources themselves modulating TLR-mediated allergic immune responses. A genetic predisposition is the basis of an individual’s susceptibility to develop allergic diseases. The involved genes influence the overall TLR responsiveness, including expression, signaling and activation. Environmental factors including the microbiome, the virome, smoking, air pollution and events in early childhood including mode of delivery, breastfeeding and upbringing modulate TLR responsiveness, which in turn, influences the development of allergies. Allergens and allergen sources, such as inhalants, injectants, contacts and food allergens, interact with TLRs either direct or indirect via their co-delivered immunomodulatory molecules (e.g., LPS). TLR, Toll-like receptor.

Based on the rapid increase of sensitization rates to pollen and food allergens, allergies nowadays represent a major global health burden (Reinmuth-Selzle et al., 2017). To understand this tremendous increase, it is of greatest interest to identify the immunological pathways involved in allergic sensitization. Environmental factors and genetic variations in TLR-related genes are not only linked to the development of allergic diseases such as asthma and allergic rhinitis (AR), but their expression also varies between individuals with allergies and those without. In this review, we aim on providing a holistic view on the involvement of TLRs, bridging the innate and adaptive immunity, in the pathogenesis of Immunoglobulin E− (IgE-) mediated allergic diseases (Figure 1), thus, incorporating general aspects such as TLR classification, ligands and signaling. Then, we will introduce the reader to the topic of allergic diseases by describing TLR expression in immune cells and tissues associated with the disease and will highlight differences in expression between allergic and non-allergic donors. We will introduce allergy-favoring and-protective immunomodulating responses that occur upon TLR activation, and how this is regulated by genetic and environmental factors. The hallmark of this review are allergen sources inducing allergy development via TLR interaction. On that foundation, we will discuss how the function of TLRs can be exploited for therapeutic purposes to treat allergies.

## TLRs: Structure, ligands, signaling and function

### TLR structure and classification

TLRs belong to the class 1 transmembrane proteins and consist of (i) ectodomains with leucine-rich repeats, for the specific recognition of PAMPs at the N-terminus, (ii) a transmembrane helix which might play an import role in TLR oligomerization of TLR2, and (iii) the Toll/Interleukin-1 receptor (TIR) domain, a C-terminal cytoplasmic domain responsible for the downstream signaling transduction (Gao et al., 2017).

Ten functional TLRs (hTLR1-10) have been described in humans and 13 (mTLR1-13) in mice, of which mTLR10 is non-functional due to a retrovirus insertion. TLRs are subdivided depending on their localization either into extracellular (TLR1-2, TLR4-6 and TLR10) or intracellular TLRs (TLR3, TLR7-9, and mTLR11-13), found within endosomal compartments (Kawai and Akira, 2010; Behzadi et al., 2021). However, in recent years, intracellular TLRs have been repeatedly shown to be expressed on cell surfaces as well (Dasari et al., 2011; Ioannidis et al., 2013; Tengroth et al., 2014). Most TLRs are homodimers, whereas TLR2 and TLR10 form heterodimers either with one another or with TLR1, TLR2 and TLR6, although the functional relevance of the heterodimerization involving TLR10 remains unclear (Behzadi et al., 2021).

### TLR ligands

**FIGURE 2:**
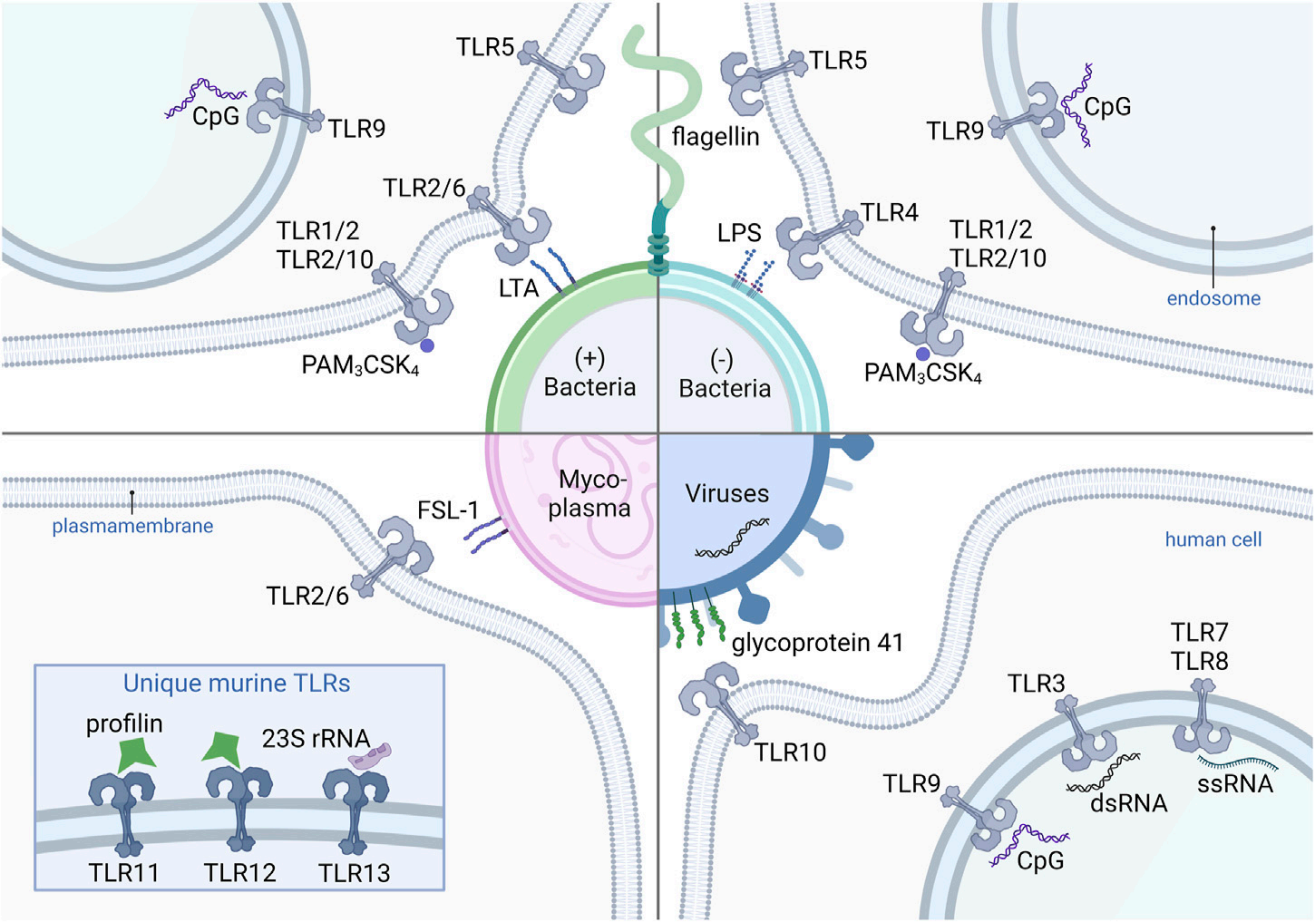
Human and murine TLRs and their corresponding ligands from different pathogens. The (+) bacteria-derived ligands LTA, PAM3CSK4, CpG and flagellin, which are recognized by heterodimers TLR2/6, TLR1/2 and TLR2/10, as well as the homodimers TLR9 and TLR5, respectively. Also (−) bacteria contain flagellin, PAM3CSK4, and CpG. LPS from (−) bacteria activates TLR4. FSL-1 recognized by TLR2/6 is a representative ligand of mycoplasma-derived compounds. Viral glycoprotein 41 binds TLR10. Virus-derived oligonucleotides such as CpG, dsRNA are recognized by TLR3 and ssRNA by TLR7 and TLR8. Mice express an unique set of TLRs including TLR11, TLR12 and TLR13, with the corresponding ligands profilins (TLR11 and TLR12) and 23S rRNA (TLR13), derived from bacteria and archaea. TLR, Toll-like receptor (+) bacteria, Gram-positive bacteria (−) bacteria, Gram-negative bacteria; CpG, CpG-oligodeoxynucleotide; PAM3CSK4, Pam3CysSerLys4; LTA, lipoteichoic acid; LPS, lipopolysaccharide; FSL-1, Pam2CGDPKHPKSF; 23S rRNA, 23S ribosomal RNA; dsRNA, double-stranded RNA; ssRNA, single-stranded RNA.

The individual receptors are highly specific for their corresponding ligands, of which the extracellular TLRs primarily recognize microbial cell wall compounds (Figure 2). The TLR2 heterodimers sense lipids, lipoproteins and lipopeptides such as the synthetic triacylated peptide PAM_3_CSK_4_ (TLR1/2), the N-terminal part of a bacterial lipoprotein, lipoteichoic acid (TLR2/6), a cell wall component of Gram-positive bacteria, and also mycoplasmal diacylated lipoproteins like FSL-1 (TLR2/6), representing the N-terminal part of the 44 kDa lipoprotein LP44 of *Mycoplasma salivarium* (Christodoulides et al., 2018). TLR2/10 heterodimers are also able to sense the majority of TLR1/2 ligands (Fore et al., 2020). TLR5 is exclusively activated by flagellin, the main constituent of bacterial flagellum and a prerequisite for bacterial mobility. Lipopolysaccharides (LPS), more precisely the containing lipid A part of Gram-negative bacteria is recognized by TLR4 and its interaction partner myeloid differentiation factor 2 (MD-2). The activation capacity of LPS varies strongly among different bacteria species. Lipid A modifying enzymes can alter, for example, the number of acyl chains or phosphate groups, which can enable host immune response circumvention. The reduction of acyl chains of the lipid A from *Bordetella pertussis* is less potent or totally lost its ability to activate TLR4, compared to the well-described hexa-acylated *E. coli* LPS (Park and Lee, 2013; Arenas et al., 2020). Although TLR10 ligands remained unknown for a long time, recently, the glycoprotein 41, a transmembrane surface protein of retroviruses such as the human immunodeficiency virus, and double-stranded RNA were described to interact with TLR10 (Lee et al., 2018; Henrick et al., 2019).

Intracellular TLRs are specialized for the recognition of bacterial as well as viral nucleic acids. TLR3 senses double-stranded RNA released during viral infections, and TLR7 and TLR8 single-stranded RNA. Bacterial and viral non-methylated, CpG-rich DNA is recognized by TLR9. Murine TLR13 recognizes 23S rRNA, a subunit of bacterial and Archean ribosomes (Kawasaki and Kawai, 2014; El-Zayat et al., 2019). Murine TLR11 and TLR12 are activated by profilin derived from *Toxoplasma gondii* (Hatai et al., 2016). To date, no activation of those receptors by pollen-derived profilins like Bet v 2, Amb a 8 or Phl p 12 has been shown.

Importantly, the recognition capacity of the TLRs is not limited to the mentioned specific ligands since an extensive number of other TLR agonists has been reported (Oliveira-Nascimento et al., 2012; Gay et al., 2014; Kawasaki and Kawai, 2014; El-Zayat et al., 2019).

### TLR signaling

The TLR signaling cascade can be divided into either i) the myeloid differentiation primary response protein 88- (MyD88-) dependent or ii) the MyD88-independent/TIR-domain containing adaptor-inducing interferon-β- (TRIF-) dependent pathway, and has currently been reviewed in more detail (Duan et al., 2022).

The MyD88-dependent pathway is associated with the downstream activation of mitogen-activated protein kinases (MAPKs) and the inducible transcription factors NF-κB, a master regulator of proinflammatory immune responses leading to the production of inflammatory cytokines (TNF-α, IL-1β, IL-6 and IL-12), or interferon regulatory factor 7 leading to the transcription of type-1 interferons such as interferon (IFN)-α (Firmal et al., 2020). Activating protein-1 is another important transcription factor, regulating the inflammatory immune response (Hu et al., 2021). This pathway is used by all TLRs, except TLR3.

TLR3-induced signaling is transmitted via the TRIF-dependent pathway, which leads to the release of type-1 IFNs such as IFN-β (Millrine et al., 2016), upon the induction of interferon regulatory factor 3. The TRIF-dependent pathway is also activated by TLR4. In contrast to TLR3 that directly recruits TRIF, TLR4 requires the TRIF-related adaptor molecule for interaction with TRIF (Lim and Staudt, 2013; Kawasaki and Kawai, 2014)**.** TLR9 can also utilize TRIF as an alternate signaling pathway (Volpi et al., 2013; Sauter et al., 2019).

## TLR expression

### TLR expression in organs associated with allergic diseases

**FIGURE 3:**
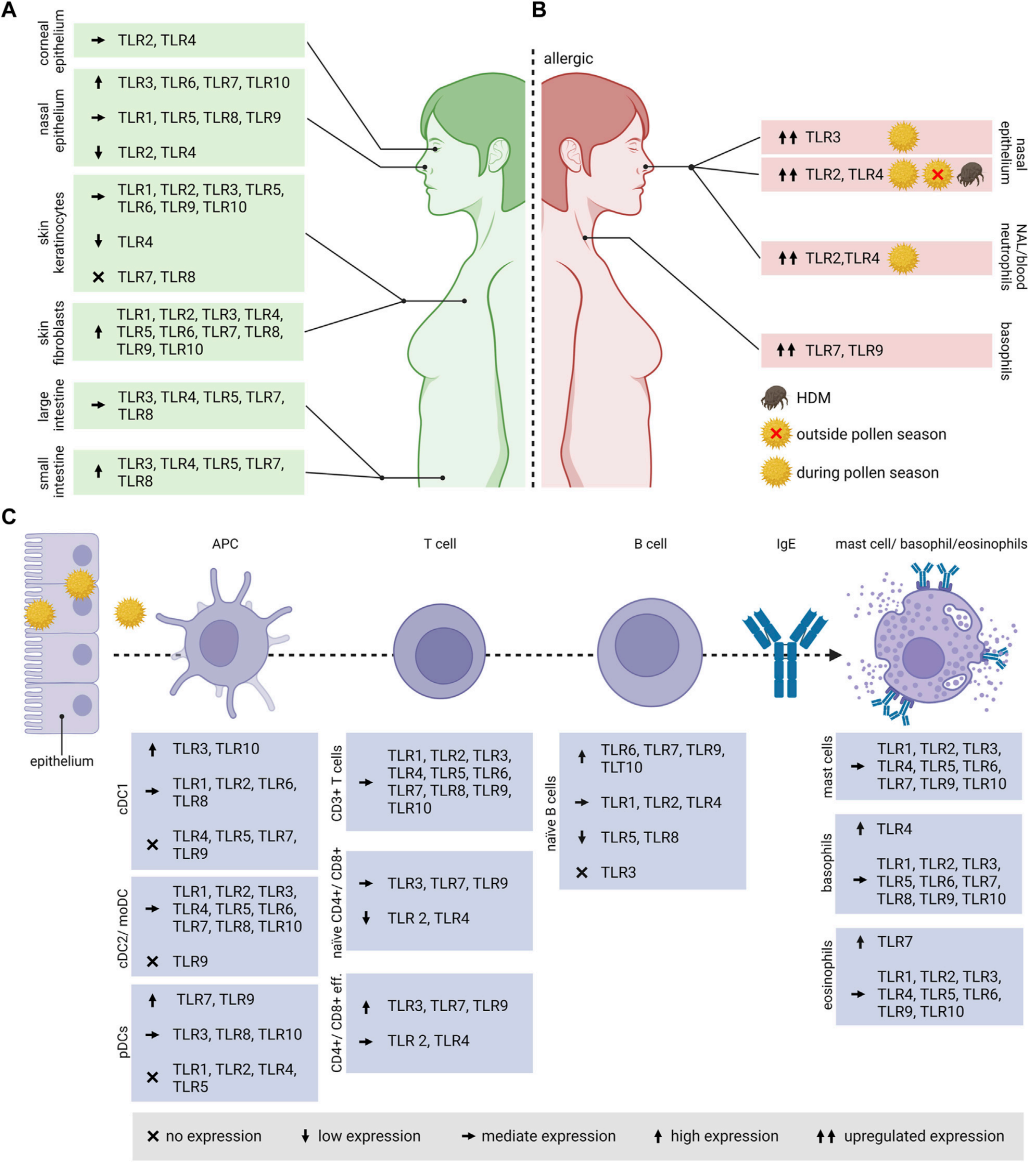
TLR expression in human cell types and tissues associated with allergic diseases. **(A)** As the route of allergen exposure varies, either via inhalation, ingestion, contact or injection, also the expression of TLRs varies in the associated organs and tissues. **(B)** In pollen allergic patients, the TLR expression differs compared to healthy individuals, and in *versus* outside the pollen season. **(C)** The expression pattern of TLRs varies in different cell types associated with the onset and progression of allergic diseases. When an allergenic source (e.g., pollen) encounters the body at the epithelium, APCs such as DCs are taking up the allergens and process them in the endolysosomal compartment for antigen presentation to naïve T cells. For Th2 polarization, the additional activation by the cytokine IL-4 is required. IL-4 and IL-13 produced by Th2 cells induces the class switch in B cells to produce allergen-specific IgE. The specific IgEs bind to the high affinity Fcε receptor I on the surface of basophils, eosinophils and mast cells. TLR, Toll-like receptor; NAL, nasal lavage; APC, antigen presenting cell; IgE, Immunoglobulin E; DC, Dendritic cell; cDC1, conventional DC1; cDC2, conventional DC2; moDC, monocyte-derived DCs; pDCs, plasmacytoid DCs; eff., effector.

Depending on the cell type, tissue region, developmental stage in life as well as health status, TLR expression profiles vary strongly (Figure 3A). Data on TLR expression concerning the upper airways including epithelial cells of the paranasal sinuses and the nasal cavity are very limited. Human nasal epithelia cells express the full range of TLRs with TLR3, TLR6, TLR7 and TLR10 being the highest expressed, whereas TLR2 and TLR4 are expressed the lowest (Renkonen et al., 2015). The nasal epithelium also expressed high levels of TLR3, TLR7 and TLR9 on the apical surface (Tengroth et al., 2014). Once activated, these receptors are upregulated. Nasal epithelial cells are hypo-responsive to LPS and produce low levels of MD-2. To avoid continuous over-activation of TLRs by a broad range of commensal and pathogenic bacteria colonizing the upper respiratory tract, several mechanisms were employed by the immune system, such as mucus production or the expression of ani-microbial proteins. In contrast, lung epithelial cells are activated by bacterial-derived LPS that further triggers DC activation (McClure and Massari, 2014; van Tongeren et al., 2015).

Little is known about TLR expression on the human corneal epithelium. On mRNA level, TLR3 and TLR4 expression was observed on the surface of such epithelial cells (McClure and Massari, 2014).

Human alveolar macrophages express the whole range of TLR mRNA, of which TLR1, TLR2, TLR4, TLR7 being the most expressed followed by lower expression levels of TLR3, TLR5, TLR6 and TLR9 (Hoppstädter et al., 2010). Whereas several studies reported TLR1-10 expression in the airway epithelium (de Waal et al., 2022), only TLR2-4 expression was described in the alveolar epithelium (Thorley et al., 2011; Wu et al., 2015). The expression in primary airway epithelia cells isolated from the human tracheobronchial epithelium varies strongly amongst the individual receptors. TLR2 and TLR6 were located mainly at the basolateral side. The expression of TLR1, TLR4, TLR5, TLR7, TLR9 and TLR 10 was predominantly limited to the luminal cell surface. Interestingly, antiviral TLR3, TLR7 and TLR9 were located mostly at the apical cell surface of the primary epithelial cells, which is contrary to the well-known localization in endosomal membranes of antigen-presenting cells. TLR1 and TLR3 was also located in the cytoplasm, but TLR8 was not detected on those cells (Ioannidis et al., 2013). The lung endothelium expresses TLR4, playing a leading role in neutrophil attraction into the lungs (Andonegui et al., 2003).

For the intestinal epithelium, the expression of TLRs is unevenly distributed amongst the small intestine and the colon. Huhta et al. observed a stronger expression of TLR3-TLR5, TLR7 and TLR8 in the human small intestine compared to the large intestine (Huhta et al., 2016). Contradictory, Price et al. showed *in vivo* by using reporter mice a much higher expression of TLR2, TLR4 and TLR5 in the colon and colon organoids and a very weak or no expression of TLR2, TLR4, TLR5, TLR7 and TLR9 in the small intestine and organoids (Price et al., 2018).

Regarding the expression of TLRs in the skin, the epidermis consists mainly of keratinocytes that express TLR1-TLR6 and TLR9-TLR10, whereas TLR4 is expressed the lowest. Skin fibroblasts on the other hand express high levels of all TLRs (Yao et al., 2015).

### TLR expression on cell types involved in the allergic immune response

TLRs play a key role in linking the innate and adaptive immune system, therefore, they are highly expressed on the respective immune cells (Figure 3C). DCs, belonging to the innate immune cells represent the most effective antigen-presenting cells.

Depending on the specific subsets and maturation stage, TLRs are differently expressed on DCs. In human peripheral blood, DCs can be divided into two different subtypes, namely, myeloid DCs, also known as conventional DCs, and plasmacytoid DCs. The former can be further divided into the rare conventional DC1 and conventional DC2. *In vitro* generated monocyte-derived dendritic cells (moDCs) are commonly used, although they do not fully resemble myeloid DCs in the blood (Collin and Bigley, 2018). Based on mRNA data, human blood CD141^+^ conventional DC1s do not express TLR4, TLR5, TLR7 and TLR9 and express high levels of TLR3 and TLR10 followed by TLR1, TLR2, TLR6 and TLR8. CD1c^+^ DC2s were reported to express all TLRs except TLR9, and is similar to the expression profile of moDCs (Hémont et al., 2013). Human plasmacytoid DCs are specialized in virus recognition based on their high expression of TLR7 and TLR9, however, lack all other surface TLRs except TLR10 (Schreibelt et al., 2010).

Like in DC subsets, TLR expression varies strongly in B and T cells. Accordingly, circulating naïve B cells show the highest levels of TLR mRNA for TLR6, TLR7, TLR9 and TLR10, moderate levels for TLR1, TLR2 and TLR4, low levels for TLR5 and TLR8, and undetectable TLR3 expression (Pettengill et al., 2016).

All of the known TLRs are expressed on T lymphocytes. Interestingly, TLR8, usually classified as intracellular TLR, is also found on cell surfaces (Dasari et al., 2011). The expression on human T lymphocytes seem to vary among subsets and their differentiation status (Nouri et al., 2021; Zhang et al., 2022). TLR levels of naïve CD4^+^ and CD8^+^ T cells are generally lower compared to activated and memory T cells. Both effector T cell subsets highly express the intracellular receptors TLR3, TLR7 and TLR9 compared to the surface receptors TLR2 and TLR4 (Hammond et al., 2010).

Although not all TLRs were found on mast cells derived from different tissue samples, on peripheral blood-derived mast cells all TLRs were identified on mRNA as well as on protein level, except TLR8 (Sandig and Bulfone-Paus, 2012). In basophils derived from PBMCs, TLR2-TLR6 and TLR9 were identified in most human donors with the highest relative mRNA level observed for TLR4, whereas mRNA encoding for TLR1, TLR7-8 and TLR10 was only identified in a few donors (Suurmond et al., 2014; Knuhr et al., 2018). Human blood eosinophils express the full range of TLRs except TLR8, with TLR7 being the highest expressed. Together with the other virus-sensing TLRs 3 and 9, its role in AR was demonstrated (Kvarnhammar and Cardell, 2012).

### Differential expression of TLRs in allergics *versus* non-allergics

So far, a difference in TLR expression between allergic and non-allergic individuals was only observed in context of inhalant allergies (Figure 3B). Patients affected by persistent AR caused by most common aeroallergens, primarily house dust mites (HDM), showed an increase in TLR2 and TLR4 mRNA and protein levels in the nasal epithelium compared to healthy controls (Cui et al., 2015). In a cohort of patients affected by birch and grass pollen, TLR2 and TLR4 was evaluated during and outside the pollen season. In nasal lavages, the intracellular expression of TLR4 on circulating neutrophils was increased, in comparison to healthy controls and patient material collected outside of the pollen season. The sampling was performed at the beginning of the pollen season (Ekman et al., 2012). An increase in nasal expression of TLR3 mRNA was also observed in birch and grass pollen allergic patients during pollen season (Fransson et al., 2005). In rats intraperitoneally sensitized with ovalbumin (OVA), an increase in TLR3 mRNA levels was observed in lymphoid tissues that correlated with the inflammatory Th2 response, suggesting the importance of TLR3 in modulating asthma. Upon blocking of TLR3, using a short-hairpin RNA plasmid, the Th2-associated IL-4 mRNA levels decreased, resulting in a reduction of total IgE in the serum (Meng et al., 2011).

The role of TLR7 in AR and asthma is often associated with an attenuation of the airway inflammation. An increase in TLR7 and TLR9 expression was observed on basophils of patients affected by AR. Patients were selected based on a positive skin prick test against *Artemisia sieversiana*, plane trees or HDM (Wang et al., 2021; Zhong et al., 2021).

## TLRs modulating allergic immune responses

**FIGURE 4:**
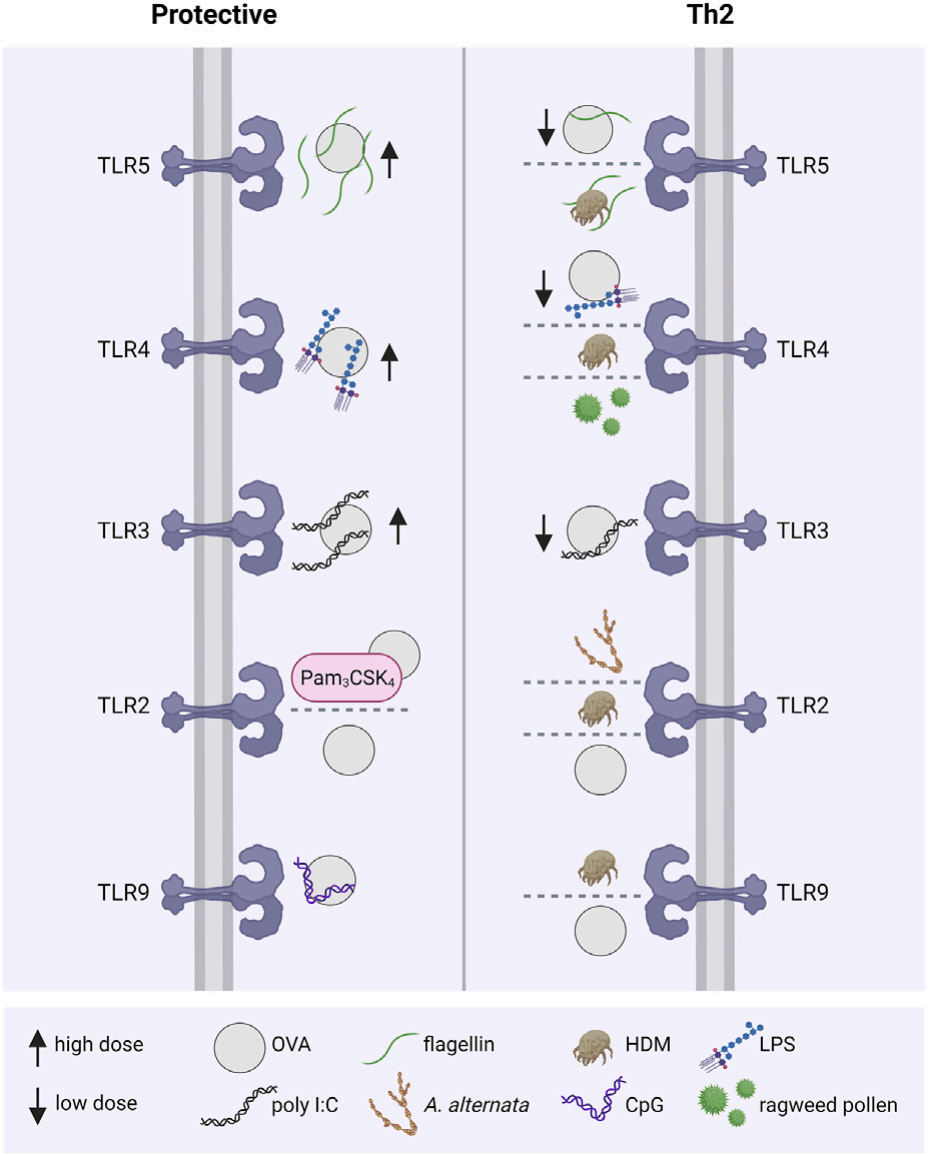
TLRs modulating allergic immune responses. The activation of different TLRs by allergen sources, including OVA, HDM, pollen or *A. alternata*, with or without the presence of TLR agonists either leads to an allergy-protective immune response or a disease-associated Th2-dominant inflammation. For example, co-administration of OVA with low doses of the TLR ligands flagellin, LPS and poly I:C resulted in allergic sensitization, whereas high doses had an opposite protective effect. Th2, T helper class type 2; TLR: Toll-like receptor; OVA, ovalbumin; HDM, house dust mite; LPS: lipopolysaccharide; poly I:C, polyinosinic:polycytidylic acid; *A. alternata, Alternaria alternata*; CpG, CpG-oligonucleotide.

TLRs play an important but controversial role in modulating the immune response. In context of allergic diseases, TLRs are either involved in inducing and exacerbating allergic inflammation and Th2 polarization, or in immunotolerance and protective responses (Figure 4).

### TLRs in the induction and exacerbation of allergic Th2 responses

The role of TLR2 in Th2 responses appears controversial. In an OVA allergy model, C57Bl/6 wild type (WT) and TLR2 knock-out (KO) mice were sensitized intraperitoneally at day 0 and challenged with inhalant OVA from day 14 to day 20. An increase in TLR2 expression in the lungs of WT mice was observed and accompanied by an enhanced infiltration of inflammatory cells and mucus production. In TLR2-KO mice the effect was milder (Jiang et al., 2017). In contrast, in an allergic asthma model using the three different allergens HDM, OVA and *Alternaria alternata*, increased TLR2 and thymic stromal lymphopoietin (TSLP) levels in the lungs of challenged mice were found. The results were validated in stimulated human bronchial epithelial cells, suggesting TSLP secretion is mediated by TLR2. An OVA challenge resulted in a decrease of CD4^+^ IL-4^+^ cells in mediastinal lymph nodes and eosinophils in bronchoalveolar lavage fluids (BALF) of TLR2-KO as well as WT mice treated with a TLR2 antagonist in comparison to WT mice, suggesting an association between TLR2 and the activation of the Th2 response (Lv et al., 2018).

Most studies usually focus on the dependency of TLR4 in Th2 responses. HDM induced DC activation through TLR4, activating Th2 response in an adjuvant-free murine model; the use of TLR4 antagonist (underacyletd form of *R. spharoides* LPS) reduced airway inflammation to the baseline level of non-sensitized mice, in terms of eosinophilia and lymphocytosis (Hammad et al., 2009). In a murine model of short ragweed pollen-induced allergic conjunctivitis, TSLP expression mediating Th2 induction occurred TLR4-dependently, based on mRNA levels of IL-4, IL-13 and IL-5 in CD4^+^ T cells. In the cervical lymph nodes, an increase in the mRNA expression of Th2 cytokines, OXA40L and TSLP receptor was observed (Li et al., 2011).

In a recent study, the TLR5 ligand flagellin exacerbated asthma in a mouse model. C57BL/06 mice sensitized with OVA were challenged with either OVA or OVA combined with flagellin. The combined challenge induced an increase in the number of eosinophils and neutrophils in BALF, and although inflammatory cytokines and chemokines, such as IL-6, IL-1β, Cxcl9, Cxcl10, Ccl3, and Ccl4, were increased, Th2 cytokines did not increase (Whitehead et al., 2020). In contrast, flagellin-dependent TLR5 activation associated with HDM-induced asthma exacerbation was demonstrated. TLR5-KO mice treated with multiple instillations of LPS-containing HDM extract (incl. the major allergens Der p/Der f 1, Der p/Der f 2, Can f 1, Bla g 2 and Alt a 1) developed a lower airway eosinophilia compared to WT mice. Upon allergen challenge only WT mice developed a strong airway hyperresponsiveness. These data were confirmed using two different extracts, and suggest that flagellin contained in the HDM extracts induces the activation of TLR5 and acts as adjuvants in priming the allergic response to indoor allergens (Wilson et al., 2012).

Less is known regarding the intracellular TLRs. In an experimental asthma model using HDM extracts, the airway hyperresponsiveness of TLR9-KO mice improved compared to the WT mice, which was accompanied by a reduction of the mRNA levels of the Th2 cytokines IL-13, IL-5, and IL-4 in the lungs. In contrast, mRNA levels of IL-17A were significantly higher in TLR9-KO mice treated with HDM extracts in comparison to WT mice. The Th2 response observed in this HDM asthma model depended on TLR9-mediated IL-2 production (Murakami et al., 2020). The same TLR9-dependency in mediating Th2 responses was demonstrated in an OVA allergy model. Here, bronchoalveolar lavage inflammatory cells including neutrophils, lymphocytes, monocytes and eosinophils were reduced in TLR9-KO mice in comparison to WT mice, treated with OVA (Zhao et al., 2020), suggesting that TLR9 plays a superior role in Th2 induction by allergens and airway inflammation (Brugnolo et al., 2003; Hatchwell et al., 2015). A major limitation of these studies is that most allergen sensitization models exploit the subcutaneous or intraperitoneal route, which is not representative for human exposure for most allergens. As outlined before, TLR expression patterns differ greatly among organs and cell types, which further influences the interpretation of these results (Kalis et al., 2003; Chen et al., 2014).

### TLRs inducing protective immune responses

Many studies emphasize the necessity of TLR signaling in allergic Th2 responses, although protective roles in allergic diseases were also discussed (Zakeri and Russo, 2018). The concrete protective roles of TLRs in counter-regulating inflammatory allergic responses can be subdivided into three distinct mechanisms: i) shifting the immune response from the pathological Th2 response towards a Th1 response or the development of immunotolerance via the induction of ii) T regulatory cells (Tregs) or iii) B regulatory cells (Bregs).

The effects of TLR activation on T cells was traditionally thought to be mediated indirectly via activated antigen presenting cells. As human T cells express TLRs, effector T cells can also be activated directly by TLR ligands. For instance, in CD8^+^ T cells deficient for IRAK-4, a protein kinase involved in TLR signal transduction, *in vitro* effector functions, such as proliferation and cytotoxicity, were impaired (Suzuki et al., 2006; Rahman et al., 2009). In an adoptive CD4^+^ T cell transfer model of inflammatory bowels disease, the transfer of MyD88-deficient T cells resulted in a reduced disease severity compared to WT cells. The opposite was observed when MyD88-deficient Tregs were co-transferred with WT CD4^+^ T cells. In that case, MyD88-deficient Tregs had a reduced capacity to mitigate disease severity compared to WT Tregs, suggesting that TLR signaling plays an important role in the function of effector T cells as well as Tregs (Fukata et al., 2008).

As allergy is defined as an exaggerated immune response towards harmless foreign substances (e.g., pollen), resulting in a pronounced IgE-mediated inflammation, it is proposed that *in utero* and during early childhood the immune system tends towards a Th2-biased response for efficient pathogen clearance. Early childhood infections and exposure to TLR ligands are believed to cause a shift towards a Th1/Th17-biased immune response, outbalancing the inherited Th2 bias (Daley, 2014). Experimentally, the modulation of allergic Th2 responses via TLR-modulated Th1 induction was shown for TLR2/1, TLR2/6, TLR3, TLR4, TLR5, TLR7, TLR8 and TLR9 (Kirtland et al., 2020). In an allergic asthma model, co-administration of the TLR2 agonist Pam3CSK4 or the TLR9 agonist CpG with OVA during the intraperitoneal sensitization protected the mice from developing allergies via inducing monocytes to secret Th1 cytokines (Huang et al., 2018) Eisenbarth et al. demonstrated that high doses of LPS (100 µg/instillation/mouse) favored Th1 responses whereas low doses (0.1 µg/instillation/mouse) favored Th2 responses in a murine intranasal sensitization model using OVA. These findings led to the hypothesis that TLR4 signaling requires a certain threshold to induce Th1 responses (Eisenbarth et al., 2002). Similar effects have been shown for TLR3 in a mouse model using OVA spiked with different doses of the viral TLR3 ligand poly I:C for intranasal sensitization. Although mice sensitized with high doses (10 µg) as well as low doses (0.1 µg) of poly I:C, both, developed a non-eosinophilic lung inflammation after the OVA challenge, only the low dose favored a Th2 response, indicated by elevated specific IgE levels. In contrast, the lung inflammation in the low-dose group was driven by a Th1 response, as indicated by elevated levels of Th1 cytokines in the BALF and elevated serum IgG2a levels. Furthermore, the development of airway inflammation depended on IL-4 and STAT6 in the low-dose group whereas on IFN-γ in the high-dose group (Jeon et al., 2007). The administration of a combination of aerosolized TLR2/6 and TLR9 ligands into mice up to 30 days before HDM-sensitization protected from allergic airway inflammation. Whether this effect was caused by a modulation towards a Th1 or a Treg response has not been investigated. Nevertheless, in an additional OVA sensitization model an increase in specific serum IgG2a, associated with a Th1 response, in mice treated with the same TLR ligands was observable (Goldblatt et al., 2022).

The other major mechanism of how TLRs provide an allergy-protective response is via the induction of Tregs, Bregs, and the inhibition of effector T cell responses. Tregs play an essential role in the protection from allergic diseases, as they counteract the exacerbation of effector T cell responses. Induction of allergen-specific Tregs is considered to be a principal mechanism in successful allergen-specific immunotherapy (AIT), as they promote the production of protective allergen-specific IgG4, which compete with IgE for the allergen binding and thus prevent the IgE-mediated allergic response (Kappen et al., 2017). As mentioned above, TLR signaling contributes to the general function and proliferation of CD4^+^ T cells regardless of their specific subsets. TLR2 stimulation, for instance, favors Treg proliferation and survival whereas TLR4 does not (Netea et al., 2004; Sutmuller et al., 2006; Nawijn et al., 2013). In OVA sensitized mice, a long-term protection from asthma was sustained by TLR2-induced Treg proliferation (Nawijn et al., 2013). Direct stimulation of TLR4 on T cells by LPS upregulates T cell adherence to fibronectin and thus inhibits cell migration towards CXCL12 via STAT3-dependent induction of SOCS3, which contributes to the termination of effector functions (Zanin-Zhorov and Cohen, 2013). Intranasal application of high doses of the TLR5 ligand flagellin during intraperitoneal OVA sensitization reduced allergic lung inflammation in mice, based on reduced airway hyperresponsiveness, BALF cell counts and OVA-specific serum IgE. This TLR5-mediated prophylactic effect depended on a different location of TLR ligand *versus* allergen application, and resulted in the generation of Tregs and tolerogenic DCs (Shim et al., 2016). Whereas co-administration of OVA and flagellin via oropharyngeal instillation, which is considered a more physiologically relevant model, rather resulted in allergic inflammation (Wilson et al., 2012; Whitehead et al., 2020). Systemic co-administration of the TLR9 ligand CpG DNA and OVA suppressed T cell expansion and the activity of cytotoxic T lymphocytes via the TRIF-dependent pathway. In contrast, subcutaneous co-administration resulted in increased OVA-specific T cell immunity (Wingender et al., 2006; Volpi et al., 2013). These controversial findings again highlight the influence of different application routes on the immunological outcome.

Besides Tregs, Bregs contribute to the maintenance of immune regulation via the secretion of anti-inflammatory cytokines IL-10 and TGF-β as well as by cell contact-dependent mechanisms like FASL-FAS ligation or PD-L1 (Mauri and Ehrenstein, 2008; Catalán et al., 2021). In the protection of allergic diseases Bregs have a specialized role due to their preferential production of blocking IgG4 antibodies upon differentiation to plasma cells (van de Veen et al., 2016). The activation of certain TLRs is capable of promoting the development of Bregs. For example, the TLR4 ligand LPS induced FASL expression on murine splenic B cells, via NF-κB signaling. These FASL^+^ B cells effectively inhibited CD4^+^ T cell proliferation. The TLR9 ligand CpG oligonucleotide failed to induce FASL^+^ B cells (Wang et al., 2017). Nevertheless, transient TLR9 stimulation of bone marrow cells with CpG DNA favored the differentiation of Bregs with protective functions against experimental autoimmune encephalomyelitis and Type 1 diabetes (Montandon et al., 2013; Korniotis et al., 2016).

## Genetic associations between TLRs and allergy

As the development of allergic diseases underlies a genetic predisposition, and since polymorphisms in TLR genes can alter their functionality, it is of most importance to dissect the genetic associations involving TLR genes in the context of allergy phenotypes, such as allergic asthma and AR (Table 1).

**TABLE 1:** Variants of Toll-like receptor genes associated with allergies.

Mapped gene	Study type	Trait(s)	SNP ID	References
***TLR1***	GWAS	allergic disease	rs11727978	Pickrell et al. (2016)
			rs148906978	Zhu et al. (2018)
			rs5743618	Ferreira et al. (2017)
			rs66819621	Johansson et al. (2019)
		asthma, allergic disease	rs6531663	Ferreira et al. (2020)
		allergic rhinitis	rs17616434	Bønnelykke et al. (2013)
			rs5743618	Ferreira et al. (2017)
		allergic sensitization	rs2101521	Hinds et al. (2013)
	Target Sequencing	asthma, bronchial hyperresponsiveness	rs4833095	Kormann et al. (2008)
			rs5743594	Kormann et al. (2008)
			rs5743595	Kormann et al. (2008)
***TLR2***	Target Sequencing	asthma, bronchial hyperresponsiveness	rs1898830	Kormann et al. (2008)
			rs3804099	Kormann et al. (2008)
			rs4696480	Kormann et al. (2008)
		allergic rhinitis	rs7656411	Chen et al. (2022)
			rs76112010	Chen et al. (2022)
			rs7682814	Chen et al. (2022)
		atopic dermatitis	rs4696480	Oh et al. (2009)
***TLR3***	Target Sequencing	asthma, bronchial hyperresponsiveness	rs3775291	Kormann et al. (2008)
***TLR4***	Target Sequencing	asthma, bronchial hyperresponsiveness	rs10759932	Kormann et al. (2008)
			rs2737190	Kormann et al. (2008)
			rs4986791	Kormann et al. (2008)
		allergic rhinitis	rs10983755	Chen et al. (2022)
			rs11536889	Chen et al. (2022)
			rs1927914	Chen et al. (2022)
			rs7873784	Chen et al. (2022)
***TLR5***	Target Sequencing	asthma, bronchial hyperresponsiveness	rs2072493	Kormann et al. (2008)
			rs5744168	Kormann et al. (2008)
			rs5744174	Kormann et al. (2008)
***TLR6***	GWAS	allergic disease	rs151067434	Kichaev et al. (2019)
		food allergy	rs3860069	Ramasamy et al. (2011)
	Target Sequencing	asthma, bronchial hyperresponsiveness	rs5743810	Kormann et al. (2008)
			rs5743789	Kormann et al. (2008)
***TLR7***	Target Sequencing	asthma, bronchial hyperresponsiveness	rs5743810	Kormann et al. (2008)
			rs179008	Møller-Larsen et al. (2008)
***TLR8***	Target Sequencing	asthma, bronchial hyperresponsiveness	rs3761624	Kormann et al. (2008)
			rs2407992	Møller-Larsen et al. (2008)
***TLR9***	Target Sequencing	asthma, bronchial hyperresponsiveness	rs187084	Kormann et al. (2008)
			rs5743836	Kormann et al. (2008)
***TLR10***	GWAS	asthma, Eczema, allergic rhinitis	rs28393318	Zhu et al. (2018)
		allergic sensitization measurement	rs28690449	Johansson et al. (2019)
		asthma, allergic disease	rs4833093	Zhu et al. (2018)
	Target Sequencing	asthma, bronchial hyperresponsiveness	rs4129009	Kormann et al. (2008)
			rs11096956	Kormann et al. (2008)
***PDCD4***	GWAS	asthma	rs6585018	Binia et al. (2013)
			rs11195360	Binia et al. (2013)
			rs1407696	Binia et al. (2013)
			rs34104444	Binia et al. (2013)

### Genome-wide association studies

Genome-wide association studies (GWAS) are a powerful tool for identifying genetic variants associated with complex diseases. GWAS involve genotyping large numbers of single nucleotide polymorphisms (SNPs) across the entire genome in a cohort of individuals with and without the disease of interest. By comparing the frequencies of SNPs between the two groups, genetic variants that are more common in individuals with the disease can be identified, providing insights into the genetic basis of the condition (Visscher et al., 2012). An association between a genetic variant and a disease in a GWAS means that the variant is statistically significantly more frequent in individuals with the disease than in those without the disease. However, an association does not necessarily imply causality (Lovinsky-Desir and Miller, 2012). GWAS have been instrumental in identifying genetic variants associated with allergies (Ferreira et al., 2011; Hirota et al., 2011; Li et al., 2012; Binia et al., 2013; Winters et al., 2019). In the context of allergic diseases, genetic variants in TLR genes have been associated with the risk of developing asthma, AR, and atopic dermatitis (Moffatt et al., 2010; Ramasamy et al., 2012; Bønnelykke et al., 2013). Recently, a genome-wide association study on severe asthmatics in a European cohort found significant associations between SNPs in the programmed cell death 4 (*PDCD4*) gene and severe childhood asthma and total IgE levels. PDCD4 is a negative regulator of TLR4. SNPs in the *PDCD4* gene could affect MYB transcription factor binding, which is linked to asthma and eosinophilia (Binia et al., 2013).

### Target sequencing

Targeted sequencing focuses on specific genomic regions of interest, allowing for a more in-depth analysis of genetic variations. This approach is particularly useful when investigating candidate genes that have been previously implicated in the pathogenesis of allergies or when seeking to validate and further elucidate the functional implications of SNPs identified by GWAS. Based on target sequencing, a protective effect for a *TLR6* as well as *TLR1* and *TLR10* SNP was described in a German population, which was characterized by the expression of Th1 cytokines and simultaneous downregulation of the Th2 cytokine IL-4 when peripheral blood mononuclear cells (PBMCs), carrying these minor allele variants were stimulated with their specific TLR ligands (Kormann et al., 2008).

The *TLR6*-*TLR1* locus is a very promising region associated with allergy development (Nilsson et al., 2014). In a Swedish study focusing on the identification of genetic variations in ten *TLR* genes of 288 patients with AR, rare variations in *TLR5*, *TLR7* and *TLR9* were identified in comparison to the general population (Henmyr et al., 2017). A case-control study including two different populations, one Swedish and one Singapore Chinese cohort, provided evidence that haplotypes in the TLR7-TLR8 gene region are closely related to AR (Nilsson D. et al., 2012). The potential to be risk genes in asthma development and related atopic disorders was previously shown based on rs179008 in *TLR7* and rs2407992 in *TLR8*, which showed the highest functional significance (Møller-Larsen et al., 2008).

Polymorphisms in *TLR2* and *TLR4* genes were discussed to influence the onset of allergic diseases, although a case-control study including atopic children with asthma, children suffering from non-atopic asthma and children with AR did not find a significant association between these two TLRs and allergic disorders, but an association with the disease´s severity was suggested (Hussein et al., 2012). However, subjects carrying one or two copies of a specific *TLR4* variant called TLR4/8551G were less responsive to endotoxins and had a nearly three-fold higher risk of being sensitized to laboratory animals and other environmental allergens than subjects with the homozygous major A allele. Since in this study people with the G variant were exposed to significantly higher endotoxin and allergen levels than people with the homozygous A variant, this result should be treated with caution (Pacheco et al., 2008). The relation between allergic diseases and a polymorphism in *TLR4* rs1927911 has recently been suggested in a pool of six birth cohorts including in total 15,299 children. It was assumed, that carriers of a minor allele of this polymorphism bear a higher risk of developing AR when reaching school age (Fuertes et al., 2013). The *TLR2* polymorphism rs4696480 was shown to be associated with the severity of atopic dermatitis (Oh et al., 2009; Potaczek et al., 2011).

In a population of Tunisian children, polymorphisms in *TLR9* and *CD14* were associated with a predisposition for asthma and atopy (Lachheb et al., 2008). Genetic associations of AR with SNPs in the TLR signaling pathway, focusing on *TLR2*, *TLR4*, and *CD14* genes were investigated in a case-control study involving 452 AR patients and 495 healthy controls from eastern China. The key finding revealed that the GT/TT genotype of the *TLR2* rs7656411 (G/T) SNP was significantly associated with an increased risk of AR. This SNP is located in the 3′near gene region, potentially affecting TLR2 mRNA transcription and gene expression. This study is the first to report the association between *TLR2* rs7656411 and AR, although this SNP has been previously associated with asthma in the Chinese population. Notably, the results of studies on *TLR2* SNPs and the risk of asthma in different populations are contradictory, and meta-analyses suggest that other SNPs, such as rs4696480 and rs3804099, are associated with asthma (Chen et al., 2022).

In the last decades it became clear that neither the environment, nor genes are standalone factors for atopy. Instead, complex multifactorial gene-gene and gene-environment interactions are likely to result in the development of allergies. For example, growing up in a farming environment and carrying the *TLR6* SNPs rs1039559T-allele and rs5743810-C-allele significantly reduced the risk of early-onset asthma, which was not observed in children not exposed to a farm environment (Lau et al., 2017). The SNP rs5743810 has previously been described to play an important role in allergic diseases, as children with a homozygous minor T-allele were more prone to AR in the age of 5–7 (Koponen et al., 2014).

## Modulation of allergy-associated TLR responses by environmental factors

According to the current state of knowledge, the environment and lifestyle strongly contribute to allergic sensitization, with TLRs playing a central role (Reinmuth-Selzle et al., 2017; Akdis, 2021; Renz and Skevaki, 2021).

### Microbial exposure

In the first decade of life, humanity is permanently exposed to microorganisms, either of pathogenic or commensal nature. This exposure starts prenatally in the mother´s womb, continues with bacterial exposure during childbirth, is introduced by breastfeeding or diet, or the consequence of housing conditions. Therefore, it is not a surprise that bacteria colonization can modulate TLR responses and shape the susceptibility for allergic diseases by providing protective mechanisms. In this regard, the hygiene hypothesis, firstly mentioned by Strachan et al., in 1989 (Strachan, 1989), nicely provides the framework for the increases in prevalence of allergic diseases in association of a decreased microbial exposure due to modern hygiene standards, and builds the foundation for other closely related hypothesis like the “old friends” (Rook et al., 2004) or the biodiversity hypothesis (Haahtela et al., 2013). The former describes the reduction of early exposure to microorganisms, co-evolving with humans and able to shape the immune system towards the induction of immune tolerance, due to increased hygiene. Within the biodiversity hypothesis the reduced diversity of environmental commensal microbiota linked to an immune disbalance, results in immune disorders like allergies.

Environmental exposure to microbes influencing the individual´s immune status and its susceptibility to develop allergic diseases starts already prenatally. The interaction of the unborn with the maternal immune system and the broad range of microbes in the umbilical cord blood, placenta, amniotic fluid and the fetal membrane has a crucial impact on allergy development in the first decade of life (Michels et al., 2018). If pregnant women living on a farm encounter a greater variety of animal species, the expression of TLR2, TLR4 and CD14 in their children is upregulated, protecting them from allergic sensitization at school age (Ege et al., 2006; Roduit et al., 2011). In a murine model using BALB/c and C57BL/6 mice, maternal intranasal exposure to the bacterium *Acinetobacter lwoffii* F78, upregulated TLR mRNA expression of TLR2, TLR3, TLR6, TLR7 and TLR9 in the lung of the mother and the production of inflammatory cytokines. This induction led to a downregulation of inflammatory cytokines such as IL-1β and TNF and TLRs in the placenta, resulting in the protection from allergic airway inflammation in the offspring, induced by intraperitoneal sensitization with OVA and aluminum hydroxide (alum) followed by an aerosolized OVA challenge. The protective effect was vanished in heterozygous offspring of mothers lacking TLR2-4, TLR7 and TLR9 (Conrad et al., 2009).

The protective effect induced by high exposure to bacteria and to endotoxins is partly explained by a phenomenon commonly referred to as endotoxin or LPS tolerance, resulting in a reduced responsiveness of TLR4 after prolonged and repeated endotoxin exposure (Seeley and Ghosh, 2017).

Vaginal delivery plays an essential role in the development of the infant´s microbiome and immune system via the contribution of TLRs. Comparing mononuclear cord blood cells collected either from caesarean sections or vaginal deliveries revealed differences in TLR responsiveness. TLR1/2-modulated IL-6 and TNF-α secretion was significantly impaired in cells obtained from caesarean section, as was the individual’s ability of clearing the airways from bacteria at the age of 12 months, accompanied by an increased risk of infantile wheezing. However, no direct correlation to atopic sensitization was observed in this Taiwanese cohort, most likely because the subjects were simply too young and no follow-up study was performed, as mentioned by the authors (Liao et al., 2017). A study including over 750,000 children between 0 and 14 could show that caesarean section is associated with an increased risk of developing asthma (Kristensen and Henriksen, 2016).

The general consensus of the scientific community is that breastfeeding is not associated with an allergy-protective effect (Mennini et al., 2021). Even though, it could be shown that breastfeeding during the initial 4 months lowers the likelihood of eczema occurrence in the first 4 years (Kull et al., 2005). The gut microbiome of breast-fed infants contains mainly *Bifidobacteria* and *Lactobacillus* bacterial strains and the milk provides innate immune factors like soluble TLR2, TLR4, and their co-receptors CD14 and MD-2. In contrast, the gut microbiome of formula-fed infants lacks these components and rather contains a broader range of microbial strains, mostly resembling the microbiome of older infants (Bäckhed et al., 2015; Lodge et al., 2015; Munblit and Verhasselt, 2016; van den Elsen et al., 2019). In a murine model using germ-free mice, the colonization of different *Lactobacillus* strains was observed to strengthen the gut epithelial barrier, resulting in protection from sensitization to the major birch pollen allergen Bet v 1, via the contribution of TLR2 (Kozakova et al., 2016).

Microbial exposure during childhood is another relevant factor. In a mouse model, the repeated administration of LPS or collected farm dust protected from the development of asthma, induced by house dust mites, and, thus, provided a first causal link between microbial exposure and protection against allergy (Schuijs et al., 2015). When comparing two different agriculture communities of the US, namely, the Amish and the Hutteries, a 4-6-fold reduced probability of asthma and allergic sensitization development was observed in Amish children. Amish and Hutteries not only share a common ancestry they also have a similar lifestyle. The big difference between both communities is their farming practice. Amish preferably live on small family farms close to their animals, using horses for transportation and fieldwork, whereas Hutteries live on highly industrialized, large communal farms. When analyzing dust samples of their homes, not only the microbial composition varied, also the endotoxin content in samples collected at Amish homes was heavily increased (6.8-fold higher). Using an *in vivo* allergic asthma model, mice were intranasally exposed to extracts derived from the collected dust and sensitized intraperitoneally with OVA together with alum followed by an intranasal OVA challenge. Amish dust extracts, containing a high LPS concentration (4,399 endotoxin units per square meter), inhibited airway hyperreactivity and provided an efficient protection against the development of asthma. In mice lacking MyD88 and TRIF, the protective effect was not observed, demonstrating the importance of TLR activation in this model (Stein et al., 2016). As outlined above the protective response mostly likely occurs due to the LPS tolerance effect and the reduced responsiveness of TLR4, resulting in a dampened immune surveillance as, e.g., by antigen-presenting cells. Other studies support the finding that children growing up in areas with high microbial exposures, like on farms, bear a lower risk of developing asthma and atopy (Ege et al., 2011; Stein et al., 2016; Frei et al., 2022).

There is strong evidence, that Treg maturation and differentiation is triggered by TLR activation in a very early stage of life. Children exposed to a farming environment, especially farm milk, have increased levels of Tregs compared to children who live in closer proximity to urban areas. It was suggested that LPS in farm milk promotes the Treg response (Lluis et al., 2014). TLR2 stimulation by peptidoglycans in mononuclear cord blood cells decreases the production of IFN-γ and anti-inflammatory cytokine IL-10 and increases the production of IL-13. Maternal allergy could thereby impair Treg development via the TLR2 pathway (Meng et al., 2016). A mouse model of colitis confirmed the involvement of TLR2, expressed on plasmacytoid DCs, and polysaccharide A, produced by the gut commensal *Bacteroides fragilis*, in the induction of Tregs (Dasgupta et al., 2014; Ramakrishna et al., 2019).

### Virome

Viral infections occurring early in life were described to promote the development of allergic diseases via disruption of the epithelial barrier integrity, as shown by viral lung inflammation induced by Influenza A, which favors Th2 polarization (Dahl et al., 2004; Looi et al., 2021). The human rhinovirus and the respiratory syncytial virus (RSV) were also described to play an important role in allergy and asthma development (Sigurs et al., 1995; Sigurs et al., 2000; Gavala et al., 2013). On the contrary, few viruses were associated with a decrease in susceptibility to allergies, as described for hepatitis A virus (Matricardi et al., 2000) or for measles. Measles, mumps and rubella vaccine administered during early childhood may offer protection against allergies for at least the first 7 years of life, and against asthma until the age of 13 (Timmermann et al., 2015). This indicates that there is an association between virus-modulated TLR activation and the exacerbation and prevention of asthma and allergies later in life. Yet, convincing evidence supporting this notion is lacking.

It is widely accepted, that surface TLRs such as TLR2 and TLR4 play a key role in viral recognition and is not just limited to intracellular ones, as recently summarized by Hatton et al. (Hatton and Guerra, 2022). Among those, RSV was the first described. Human airway epithelial cells, infected with RSV, upregulated TLR4 surface expression and, in turn, increased TLR sensitivity for LPS-dependent production of inflammatory cytokines, which was suggested to modulate airway inflammation (Monick et al., 2003). Thus, it has been hypothesized that the fusion protein F of RSV interacts with TLR2 and TLR4, but the exact involvement of TLRs in RSV recognition needs further investigation (Kurt-Jones et al., 2000). TLR2 participates in identifying several viruses, such as the human immunodeficiency virus (Nazli et al., 2013), Epstein-Barr-virus (Zheng et al., 2014), and many others, whereas TLR4 was suggested to be involved in the recognition of Ebola virus (Iampietro et al., 2018) or human papillomavirus (Yan et al., 2005).

Intranasal sensitization of murine neonates with single-stranded RNA, inducing TLR7 activation during their first day of life, led to enhanced lung and airway eosinophilia, a higher IL-13 mRNA level and an enhanced mucus production when compared to weanlings receiving the same treatment. These data suggest that the time of occurrence of viral infections throughout lifetime is crucial in modulating the immune system towards a higher susceptibility for allergic diseases (Phipps et al., 2009).

### Smoking

The association between smoking and allergies remains contradictory. Although an association between smoking and chronic rhinitis was observed, no connection was found between smoking and AR (Hisinger-Mölkänen et al., 2018). Even a protective effect of tobacco smoke concerning allergic sensitization was discussed (Shargorodsky et al., 2015). Regardless of that, children exposed to secondhand tobacco smoke by their smoking parents suffer from a higher risk of allergic sensitization to food and inhalant allergens at the age of 4–16 years (Thacher et al., 2016). A meta-analysis on the influences of (secondhand) smoking on allergy development further support these findings (Saulyte et al., 2014). The exact underlying mechanisms and the contribution of TLRs remains unclear; however, a possible suggestion is that tobacco smoke negatively affects the skin barrier integrity, allowing the penetration of allergens.

Regarding the involvement of TLRs, maternal smoking during pregnancy resulted in a reduced responsiveness of TLRs in the infants. This was investigated using cord blood mononuclear cells that were stimulated with different TLR ligands. In neonates of smoking mothers, a significantly weaker TNF-α, IL-6 and IL-10 response was observed upon stimulation with the TLR2 activator Pansorbin. Stimulation with poly I:C or LPS, the agonists of TLR3 and TLR4, respectively, showed damped TNF-α responses. Binding of the TLR9 ligand CpG C led to weaker IL-6 responses in the infants of smoking mothers. Interestingly, the activation with CpG B showed higher IFN-γ levels, associated with a Th1 response (Noakes et al., 2006). As mentioned in the previous sections, the correct expression of TLRs is crucial for an appropriate immune response triggered by pathogens and environmental components. The decreased responsiveness and decrease of certain cytokines may result in an immune imbalance favoring Th2 responses.

In contrast to this reduction in TLR responsiveness, the stimulation of a human bronchial epithelial cell line with cigarette smoke extracts amplified LPS-binding and the activation of TLR4 leading to a downstream release of IL-8. This cytokine is a potent chemoattractant for neutrophils, and neutrophil infiltration into the airways contributes to airway diseases (Pace et al., 2008).

### Air pollution

Similar to cigarette smoke, traffic-related air pollutants are another reported source promoting allergic sensitization (Brauer et al., 2007; Gruzieva et al., 2012; Bowatte et al., 2015; Brandt et al., 2015; Codispoti et al., 2015; Jung et al., 2015; Murrison et al., 2019), although the involvement of TLRs in this process is barely discussed. Exposure to particulate matter, such as diesel exhaust particles, and ozone alter TLR signaling because these pollutants, via acting as TLR ligands, reduce the recognition of PAMPs and DAMPs (Naclerio et al., 2020). Ozone as a highly reactive molecule, has the ability to degrade hyaluronan to lower molecular fragments that directly interact with TLR4 and activate the TLR4-MYD88 pathway and pulmonary DCs, thus, promoting allergic sensitization (Hollingsworth et al., 2010). TLR2 is also discussed to play a key role in the inflammatory response caused by ozone (Lucas and Maes, 2013).

## Interaction of allergens and allergen sources with TLRs

Examples involved in TLR activation can be found in allergen sources sensitizing via all different routes of exposure, including ingestion and inhalation. Even for injectants such as insect venoms an indirect role was described. The direct interaction between allergens and TLRs appears to occur solely between clearly defined allergen families, possessing either (i) specific properties for lipid-binding and shuttling of TLR ligands or (ii) indirect properties by generating DAMPs. The interaction of allergens with TLRs has mostly—if not only—been described for TLR4, and the endotoxin LPS seems to play a superior role in this regard. Since many allergens belong to protein families with lipid-binding properties including 2S albumins (Guéguen et al., 1996; Burnett et al., 2002), non-specific lipid-transfer proteins (nsLTPs), lipocalins and Bet v 1-like proteins (Jappe et al., 2019), it was obvious for researchers to focus on either TLR2 or TLR4, recognizing bacteria-derived lipoproteins and LPS, respectively. Lately, growing interest emerges for understanding the mechanistic link between immunogenicity and allergenicity of allergens in dependency of their ability to interact with TLRs. Hereinafter, the capacity of allergens and allergen sources such as HDM feces, animal dander, pollen, insect venoms and foods in inducing TLR signaling either directly or indirectly by binding to co-factors or ligands will be described (Table 2).

**TABLE 2:** Allergens and allergen sources interacting with Toll-like receptors.

Exposure	Allergen source/allergen		Involved TLR	Association to allergic response	References
Inhalation	*Dermatophagoides pteronyssinus* (European house dust mite)	Der p 2	TLR4	Increase in lung inflammation, TLR4-KO did not develop Th2 inflammation	Trompette et al. (2009)
				Decrease in Th2 inflammation	Hammad et al. (2009)
		Der p 13	TLR2	Increase in lung inflammation	Satitsuksanoa et al. (2016)
	*Aspergillus oryzae* (Rice mold)	PAO	TLR4	Increase in lung inflammation, Th2 favoring environment	Millien et al. (2013); Cho et al. (2018)
	*Felis domesticus* (domestic cat)	Fel d 1	TLR4	n.d	Herre et al. (2013)
			TLR2	n.d	Herre et al. (2013)
	*Canis familiaris* (domestic dog)	Can f 6	TLR4	n.d	Herre et al. (2013)
	*Ambrosia artemisiifolia* (ragweed)	Ragweed pollen extracts	TLR4	allergic airway inflammation	Hosoki K et al. (2016a)
	*Betula verrucosa/pendula* (birch)	Birch pollen extract	TLR4	DC activation	Pointner et al. (2021)
				initiate airway inflammation	Shalabay et al. (2013)
		Bet v 1	TLR4	n.d	Soh et al. (2019); Rocha et al. (2016)
Inhalation and Ingestion	*Parietaria judaica* (Pellitory of the wall)	Par j 1	TLR4	n.d	Bonura et al. (2013)
	gluten-containing cereals	ATIs	TLR4	Activation of dendritic cells, macrophages, and monocytes; airway hyperresponsiveness and intestinal/lung allergic inflammation	Junker et al. (2012); Cuccioloni et al. (2016); Bellinghausen et al. (2019)
Injectants	insect venoms	Hyaluronidases	TLR4, not TLR2	DC activation; enhanced T cell proliferation; HMGB-1 is released upon cell damage, production of IL-18, allergic airway inflammation	Termeer et al. (2002); Galbiati et al. (2014); Ma et al. (2015); Bellussi et al. (2017)
			TLR2, not TLR4	immunostimulatory activity	Scheibner et al. (2006)
Allergic contact dermatitis	metals	Nickel	TLR4	innate inflammation; TNF secretion, ear thickness and leucocyte skin infiltration	Schmidt et al. (2010)
		Cobalt and palladium	TLR4	Activation of innate immune cells	Raghavan et al. (2012)

### Inhalation: House dust mites

Several allergens derived from the European HDM *Dermatophagoides pteronyssinus* display lipid-binding capacities, which have been associated with severe allergic reactions and respiratory symptoms such as asthma (Jappe et al., 2019).

The contribution of TLRs in allergic diseases was initially described by the finding, that recombinant Der p 2, one of the major HDM allergens, can promote TLR4 signaling by functional mimicking MD-2. This small protein is essential for the signaling pathway of TLR4 by binding to the ectodomain forming a TLR4-MD-2 heterodimer able to recognize LPS, a common bacterial contaminant in allergen sources (Trompette et al., 2009). In the presence of the TLR4 cofactor MD-2, Der p 2 was shown to increase the LPS (10 ng/mL)-induced activation of TLR4 *in vitro*. This TLR4-dependent auto-adjuvant activity of Der p 2 was also verified *in vivo*, since WT and MD-2-KO mice, developed allergic asthma when sensitized and challenged with rDer p 2 in presence of 0.026 pg LPS, shown by a significant increase of eosinophils in the BALF, lymphocytosis, mucous metaplasia and increased plasma IgE concentrations. Unfortunately, no specific IgE data were shown, demonstrating limitations of this study. In TLR4-KO mice, rDer p 2 along with a low dose of LPS as well as whole HDM extracts containing 1.05 ng LPS per mg extract, did not induce airway Th2 inflammation in comparison to WT mice. Both *in vivo* studies did not include a sensitization protocol with Der p 2 in the absence of LPS, but *in vitro* data showing that rDer p 2 in the absence of LPS did not induce TNF-α, IL-12/23p40 and IL-6 in mouse peritoneal macrophages indicating that the presence of LPS is indeed essential in facilitating TLR4-dependent activation of macrophages (Hammad et al., 2009; Trompette et al., 2009). In contrast, Der p 2 sensitization via the skin resulted in a protective role of TLR4 compared to sensitization via the bronchial epithelium. More precisely, TLR4 deficiency in mice that were epicutaneously sensitized with 100 μg endotoxin-reduced rDer p 2 elicited an enhanced Th2 response and IgE antibody production in contrast to WT mice, although this effect was not significant. These findings emphasize the diverse role of a given receptor depending on the contact site and leading to the suggestion that other receptors like TLR2 or inflammasome proteins such as NLRP3, abundant in the skin could be involved in the compensation of the lack of functional TLR4. Notably, in comparison to studies performed on the contribution of TLR4 activation in sensitization via the respiratory tract, much higher concentrations of rDer p 2 were used to sensitize via the skin maybe due to reduced or non-existent expression levels of TLR4 in the skin (Stremnitzer et al., 2014; Vaure and Liu, 2014). The MD-2/TLR4 complex mimicry was proposed to constitute a molecular basis for the allergenicity of group 2 mite aeroallergens, like Der f 2 (*Dermatophagoides farinae*) as they all possess the lipid-binding domain shared with MD-2. Der p 2 homologs, of the NPC2 protein family, are also found in mammals such as cats and dogs, although due to a low amino acid sequence identity it remains unclear if these proteins possess the same MD-2-mimicking function (Khurana et al., 2016; Zhu et al., 2021).

Other HDM allergens such as Der p 7 and Der p 13 contain hydrophobic cavities that enables the allergens to bind lipid cargos, which seem to be involved in triggering TLR pathways (Satitsuksanoa et al., 2016; Jappe et al., 2019; Jacquet and Robinson, 2020). Der p 7 was reported to bind the bacterial lipopeptide, polymyxin B, known to bind and neutralize LPS, although only with weak affinity (Mueller et al., 2010). Its homolog of the tropical mite *Blomia tropicalis*, Blo t 7, induced IL-8 and GM-CSF secretion in airway epithelial cells in a TLR2-dependent manner and the recombinant allergen was shown to bind selectively to cis-parinaric acid, a polyunsaturated fatty acid (Soongrung et al., 2018). For Der p 13, a selective binding of fatty acids and hydrophobic ligands was reported. In an *in vitro* study using human bronchial epithelial cells, Der p 13 was shown to facilitate the production of IL-8 and GM-CSF and that this activation of airway epithelial cells was TLR2-MyD88-NF-κB- and MAPK-dependent. Interestingly, Der p 13 triggers the production of these cytokines just in an intact form, indicating that Der p 13 indeed enables the transfer of fatty acid/lipid to TLR2 or a TLR2 co-receptor (Satitsuksanoa et al., 2016).

### Inhalation: Fungi

Allergenic sources-derived proteinases, especially cysteine proteases such as the major HDM allergen Der m 1 (*Dermatophagoides microceras*) (Hu et al., 2022) but also fungal proteinase from *Aspergillus oryzae* (PAO) (Landers et al., 2019), were observed to indirectly activate TLR4 by generating byproducts of proteolytic degradation acting as DAMPs. In such case, the TLR4 pathway is activated by cleavage products of fibrinogen, a protein regularly circulating through the blood stream of vertebrates and highly relevant in blood-clotting (Hodgkinson et al., 2008). Two research groups investigated the role of TLR4 in fungal protein allergen PAO-induced allergic airway inflammation *in vivo* (Millien et al., 2013; Cho et al., 2018). Intranasal administration of PAO elicited characteristic features of asthma in WT mice, whereas TLR4-KO mice exhibited reduced asthma symptoms but sustained IL-4 levels. The dependency on TLR4 to induce allergic lung inflammation, nonetheless, seemed to be equivalent for non-protease activity-containing allergens such as OVA. Incubation of fibrinogen with PAO or thrombin generated fibrinogen cleavage products that are able to induce the expression of IL-13Rα1 and MUC5AC in *ex vivo*-treated airway epithelial cells, which was also demonstrated to be relevant *in vivo* (Millien et al., 2013). Moreover, generated fibrinogen cleavage products caused an increase in Th2-favoring PD-L2 DCs in mediastinal lymph nodes after intranasal immunization with PAO, by stimulating mast cells to produce IL-13 in a TLR4-dependent manner (Cho et al., 2018). Alternatively, a consequence of the TLR4 activation is the production of intracellular reactive oxygen species acting as second messengers of cellular stress in the process of allergic sensitization (Zhang et al., 2018; Abu Khweek et al., 2020).

### Inhalation: Animal dander

Allergenicity of animal dander has also been associated with immunostimulatory effects resulting from the binding of certain allergens such as uteroglobin and lipocalins to lipoteichoic acid or LPS, the microbial agonists of TLR2 and TLR4, respectively (Herre et al., 2013). The major cat dander allergen, rFel d 1 (0.5 ng of LPS per mg protein), was shown to enhance LPS-mediated activation of TLR4 in reporter cell lines by approximately 15-fold and to enhance lipoteichoic acid-induced activation of TLR2, but to a lesser extent. In murine DCs, the production of the pro-inflammatory cytokine TNF-α resulting from an activation of TLR4 or TLR2 by LPS, lipoteichoic acid or other lipopeptides was increased by rFel d 1. Interestingly, the authors did not find a formation of a complex between recombinant Fel d 1 and TLR4/MD-2, which is in contrasts to Der p 2, indicating that Fel d 1 enhances TLR4 signaling through a different mechanism. Instead, Fel d 1 was suggested to facilitate LPS transfer resulting in TLR4 activation, since direct interaction of Fel d 1 to LPS was shown via LPS pull-down experiments (Herre et al., 2013).

The dog dander allergen, Can f 6 of the lipocalin family, contains a hydrophobic binding pocket for small lipophilic molecules and was also described to enhance LPS-induced activation of TLR4 in primary macrophage-like cells (Herre et al., 2013). However, in contrast to the uteroglobin Fel d 1, effects mediated by the lipocalin Can f 6 occurred MD-2-independently (Madhurantakam et al., 2010; Nilsson O. B. et al., 2012). These results rise the question if other lipocalin allergens, such as horse Equ c 1/2, sharing structural homologies could also function as TLR activators during allergic diseases (Jensen-Jarolim et al., 2016).

### Inhalation: Pollen

Pollen represents a major source of inhalant allergens affecting up to 40% of patients suffering from respiratory allergies in Europe (Biedermann et al., 2019). Besides the allergens, pollen contain a complex matrix that can be subdivided into the intrinsic part, which includes proteins, lipids, and metabolites inherent to the pollen, and the extrinsic part, which is mostly influenced by the pollen microbiome consisting of Gram-positive as well as Gram-negative bacteria (Ambika Manirajan et al., 2016; Obersteiner et al., 2016; McKenna et al., 2017; Pointner et al., 2020). The growing knowledge about the presence and composition of the pollen microbiome, raised the question if it possesses immunostimulatory capacities. Pollen-associated bacteria are diverse, plant species-specific and their composition is influenced by environmental factors such as air pollution (Obersteiner et al., 2016). To bring comprehension into the pollen´s microbial inhabitants, Manirajan et al. compared diversity, structure, and colonization pattern of four different pollen using cultivation-dependent and -independent methods. The study confirmed species-specific features and identified *Proteobacteria* as being the most abundant phyla in the pollen, followed by *Actinobacteria, Firmicutes* and *Bacteroitedes* (Ambika Manirajan et al., 2016). In this regard, the pollen-associated microbiome obviously provides TLR agonists (i.e., LPS contaminations in pollen extracts), but intrinsic pollen-derived TLR ligands are merely described. Studies commonly limit their investigation to the role of TLRs induced by whole allergenic extracts and rarely complete the identification and characterization of the responsible candidate compounds (Boasen et al., 2005; Hammad et al., 2009; Kamijo et al., 2009; Li et al., 2011; Shalaby et al., 2012; Shalaby et al., 2013; Hosoki et al., 2014; Hosoki et al., 2016a; Hosoki et al., 2016b; Li et al., 2016). The complex, heterogenous and highly variable composition of allergenic sources (Bashir et al., 2013; Pointner et al., 2020) as well as the remarkable chemical and structural variety of TLR ligands associated with a diverse activity spectrum might be the main reason for this limitation (Taylor et al., 2007; Lorne et al., 2008; Erridge, 2010; Schmidt et al., 2010; Manček-Keber and Jerala, 2015). Hosoki et al. suggested the existence of pollen-derived intrinsic TLR4 agonists based on the finding that ragweed pollen extracts (containing low endotoxin concentration) induced a TLR4-dependent CXCR2-mediated neutrophil infiltration in BALF in course of allergic airway inflammation induced by intranasal instillation *in vivo* (Hosoki et al., 2016a). In an additional study, the mechanism was not attributable to the effect of LPS as the response occurred independently of CD14 (Hosoki et al., 2016b). In this regard, we have recently shown that birch pollen extracts (BPE) may contain, in addition to microbiome-derived LPS, intrinsic TLR4 agonists involved in DC activation and subsequent Bet v 1-specific T cell stimulation in an *in vitro* co-culture model with pulsed DCs. However, the exact molecular structure of this TLR4 ligand is still under investigation (Pointner et al., 2021). By examining the possible interrelation between the TLR4-TRIF pathway and oxidative stress in the different developmental stages of birch pollen-induced allergic airway disease *in vivo*, Shalaby et al. demonstrated that oxidative stress occurs during allergic sensitization, as evaluated by measuring BPE-specific serum IgE, and by allergen challenge (Shalaby et al., 2013). The involvement of the TLR4 pathway was different in the two phases of the allergic disease. While TLR4 was necessary to initiate airway inflammation shown by reduced neutrophil, lymphocyte, and eosinophilia recruitment as well as IL-4 and IL-10 cytokines in the lungs in TLR4-KO or -antagonized mice immunized with BPE, compared to mice treated with PBS, the downstream adaptor TRIF had the contrary effect of dampening the inflammatory response induced by BPE airway exposure. To examine the contribution of pollen-intrinsic NADPH oxidase in this process, a heat-inactivated form of BPE was administered to the mice, which did not alter the induced allergic sensitization nor the challenge-elicited airway inflammation and hyperresponsiveness. Although a more specific method could have been used to inhibit the NADPH oxidase activity to exclude the loss of other unrelated biological activities within the extract, these findings suggested that pollen-derived components other than NADPH oxidases may be involved in airway hyperresponsiveness, possibly cysteine proteases as they are able to produce reactive oxygen species. Different results regarding the role of NADPH oxidase were reported *in vivo* in allergic models induced by ragweed pollen (Boldogh et al., 2005; Dharajiya et al., 2007). Still, these latter mouse models were performed using the intraperitoneal route causing a systemic response, thus, rendering a direct comparison difficult. Although the major birch pollen allergen Bet v 1 binds various natural hydrophobic ligands, including pollen-derived flavonoids and phytohormones, within its hydrophobic pocket, an LPS-binding activity could be excluded with high certainty (Soh et al., 2019; Aglas et al., 2020). In general, Bet v 1 shows a promiscuity in terms of ligand binding (Mogensen et al., 2002; Chruszcz et al., 2021). Of interest, the major allergen was reported to bind palmitic acid, a saturated fatty acid, also found in the birch pollen, and claimed to interact and activate TLR4 (Ischebeck, 2016; Rocha et al., 2016). The functional significance of this allergen-TLR4 agonist interaction on TLR4 activation has not been investigated yet. As Bet v 1, most purified allergens do not have an auto-adjuvant activity and are incapable of triggering *in vivo* Th2 responses and allergic sensitization by themselves. Such allergens require the presence of their original allergenic context containing essential adjuvants and Th2-favoring factors depending on the allergenic source (Parviainen et al., 2013; Wimmer et al., 2015; Aglas et al., 2018; Araujo et al., 2020). However, the exact role of TLR4 signaling and of *per se* non-allergenic pollen-derived TLR ligands in facilitating Th2 polarization and allergic sensitization to birch pollen, remains to be elucidated.

### Inhalation/ingestion: nsLTPs

Prevalent in plants, including pollen and fruits, nsLTPs constitute an important inhalant and food allergen family, especially in middle Europe (Scheurer et al., 2021; Gonzalez-Klein et al., 2022). A predominant weed pollen allergen source found in the Mediterranean region, are Parietaria pollen, belonging to the Utricaceae family (D'Amato et al., 2007). The major allergen of Parietaria pollen, Par j 1, contains an LPS-binding region. In contrast to the majority of allergens that positively regulate the TLR4 activity, the isoform Par j 1.0101 was demonstrated to inhibit LPS-triggered responses *in vitro*, as the secretion of IFN-γ was reduced in murine splenocytes and human PBMCs stimulated with different combinations of LPS and Par j 1.0101 (Bonura et al., 2013). However, the study misses the proof of a direct involvement of the TLR4 pathway, only highlighting an endotoxin inhibitory capacity. However, it remains unclear how the endotoxin inhibitory capacity contributes to the development of allergic sensitization and whether this property is also shared by other members of the nsLTP family of allergens.

### Inhalation/ingestion: Alpha-amylase/trypsin inhibitors (ATIs)

Alpha-amylase/trypsin inhibitors are well-categorized inhalant and food allergens present in gluten-containing cereals such as wheat, barley, rice, and rye. They are known to be involved in intestinal inflammatory diseases, including coeliac disease and food allergy, but also in respiratory allergies as the so-called backer’s asthma, a prevalent occupational disease (Geisslitz et al., 2021). Like nsLTPs, ATIs belong to the prolamin superfamily of allergens. In rye, for example, the ATI Sec c 38, was identified as allergen (García-Casado et al., 1995). Especially in wheat but also in related cereals, ATIs were reported to display strong innate immunostimulatory properties in human and murine DCs, macrophages, and monocytes. The proposed mechanism involves the TLR4 signaling in complex with the cofactor MD-2 or in the presence of CD14 (Junker et al., 2012; Cuccioloni et al., 2016). Experimentally, the wheat ATI was shown to bind to TLR4 with nanomolar affinity. Bellinghausen et al. demonstrated the adjuvant activity of ATI in an *in vitro* co-culture model with autologous DCs and T cells derived from birch or grass pollen allergic donors (Bellinghausen et al., 2019). Additionally, in a humanized mouse model, ATIs caused an exacerbation of intestinal and lung allergic inflammation, and of airway hyperresponsiveness in a TLR4-dependent manner by using an anti-human TLR4 blocking antibody. Another *in vivo* study confirmed these findings by showing that an ATI-containing diet prompted a TLR4-dependent exacerbation of allergic airway and gut inflammation in comparison to mice fed with ATI- or gluten-free nutriments (Zevallos et al., 2019). Remarkably, ATI nitration, possibly caused by peroxynitrite formation or by exposure to environmental air pollutants, further enhanced the TLR4-mediated immunostimulatory response induced by unmodified ATIs in different *in vitro* experimental settings. In an autologous co-culture model, DCs pulsed with nitrated ATI significantly triggered T cell proliferation as well as IL-5, IL-6, IL-10, IL-13 and IFN-γ cytokine production compared to unmodified ATI-treated DCs (Ziegler et al., 2018).

### Injectants: Hyaluronidases

Hyaluronidases are major allergens found in insect venoms, such as bee (Api m 2) or wasp (Pol a 2) venom (Marković-Housley et al., 2000). Via their enzymatic activity, hyaluronidases are able to degrade hyaluronic acid secreted by epithelial cells at the local exposure sites to low molecular weight hyaluronic acid (LMWHA) soluble factors (Burzyńska and Piasecka-Kwiatkowska, 2021). This activity is suggested to facilitate the dispersion of venom-contained toxins by increasing the connective tissue permeability and by reducing the body fluids viscosity. Fragments of hyaluronan mediated DC activation, represented by upregulated MHCII and TNF-α production via TLR4 but not TLR2, as demonstrated in TLR4-and TLR2-deficient DCs. Moreover, LMWHA-stimulated DCs efficiently enhanced T cell proliferation in a co-culture model compared to stimulation with high MWHA (Termeer et al., 2002). Scheibner et al. claimed opposing results, showing that rather TLR2 than TLR4 is involved in the immunostimulatory activity of LMWHA (Scheibner et al., 2006). To our knowledge, direct interaction studies between these allergens and TLR4 do not yet exist. Of note, another DAMP, the so-called high-mobility group box protein 1 (HMGB-1), which can indirectly be released by damaged epithelial cells upon exposure to allergenic sources, was demonstrated to induce the TLR4-dependent production of IL-18 by skin epithelial cells (Galbiati et al., 2014). HMGB1 was demonstrated *in vivo* to regulate allergic airway inflammation in several models of allergic diseases suggesting an association between the innate immune stimulation and the Th2 inflammation (Straub et al., 2010; Ma et al., 2015; Bellussi et al., 2017).

### Allergic contact dermatitis: Nickel

Allergic contact dermatitis (ACD) in humans is mostly triggered by cutaneous exposure to nickel, a ubiquitous metal often found in cosmetic products and jewelries. Ingestion of dietary product containing nickel can also induce ACD via systemic reaction. To date, the prevalence of ACD to nickel is estimated to reach up to 19% of adults and up to 10% in children among the general European population, with an approximatively 4-fold higher incidents in females than males (Ahlström et al., 2019). The skin barrier function plays a crucial role in effectively limiting the entry of external antigens, and thus is central in the pathophysiology of ACD. In this regard, two specific mutations in the *fillagrin* gene (R501X and 2282del4), leading to an altered expression, were associated with ACD to nickel (Novak et al., 2008). Different than IgE-mediated type I hypersensitivity, ACD to nickel is a cell-mediated delayed hypersensitivity. Beside acting as an hapten (Chipinda et al., 2011), a low molecular weight substance able to elicit an immune response upon binding to a protein carrier but not on its own, nickel was demonstrated to trigger innate inflammation via TLR4 activation in cellular *in vitro* assays (Schmidt et al., 2010). The authors excluded possible effects induced by LPS contaminations via the limulus amebocyte lysate assay and by using the LPS-scavenger polymyxin B. The metal possesses the ability to directly interact with hTLR4 through two particular histidine residues, H456 and H458. Taking advantage of *in silico* structural modeling of potential binding sites, the nickel-TLR4 interaction was proposed to lead to the coordination of two Ni^2+^ ions followed by dimerization and consequent activation of the receptor. In contrast, mTLR4 cannot be activated by nickel, because it lacks these histidine residues. By generating mice transgenic for hTLR4, the researchers investigated nickel-induced ACD *in vivo*. Macrophages from hTLR4-transgenic mice secreted TNF after nickel treatment compared to macrophages not bearing the hTLR4. Nickel exposure induced ear thickness and leucocyte infiltration into the skin of sensitized hTLR4-transgenic mice in contrast to mTLR4 or TLR4-deficient mice. It therefore seems that nickel has an auto-adjuvant feature by promoting innate immune stimulation. Similar to nickel, cobalt and palladium were also described to trigger TLR4 signaling on innate immune cells such as DCs (Raghavan et al., 2012; Rachmawati et al., 2013).

## TLRs in treatment of allergic diseases

Despite the vast involvement of TLRs in immunotolerance induction, their potential in allergy treatment has not yet fully been uncovered. This chapter outlines novel treatment approaches for allergic diseases and, thus, focuses on clinical trial data. Current therapeutic strategies making use of TLR responses include i) TLR ligands or ii) TLR-ligand allergen fusion proteins, both using TLR-mediated adjuvant functions in guiding the adaptive immune response in the course of AIT, iii) the inhibition of TLRs, or even preventive treatment strategies targeting TLR activation via ligands iv) or indirectly by altering the microbiome via the diet or probiotics (v).

TLR ligands are not only able to shift the immune balance, they also accelerate DC activation and antigen uptake (Deifl et al., 2014). The potential of TLR agonists in AIT has been reviewed in detail, yet the TLR4 agonist monophosphoryl lipid A (MPLA), a detoxified version of bacterial LPS, represents the only TLR agonist used in AIT products available on the European market (Jensen-Jarolim et al., 2020; Kirtland et al., 2020; Jensen-Jarolim et al., 2021). Although comparative longitudinal studies between MPLA and other market-available AIT adjuvants such as the commonly used alum are lacking, there are indications that MPLA is as efficient and safe as the alternatives (Kopp et al., 2019; Zielen et al., 2019).

In contrast to TLR4, also agonists for the intracellular TLR7 and TLR9 were screened regarding their efficacy to alleviate allergic symptoms in clinical trials. The main idea is to overcome the allergic inflammatory Th2 response via inducing a viral or bacterial infection-associated Th1 immune response. In this regard, the TLR7 agonist AZD8848 was designed for topical airway application. In birch and grass pollen AR patients, the repeated intranasal administration of AZD8848, once weekly for 5–8 weeks, resulted in reduced nasal symptoms after a titrated nasal challenge with either grass or birch pollen. The treatment was generally well tolerated, however, led to the local and systemic induction of the type-1 cytokine IFN-γ, and was accompanied with temporary flu-like symptoms (Greiff et al., 2012; Greiff et al., 2015). In patients with mild and moderate allergic asthma, intranasal administration of AZD8848 significantly reduced asthma symptoms 1 week post-treatment when compared to placebo (Leaker et al., 2019). Four weeks post-treatment this effect was no longer significant. In contrast, for another TLR7 agonist, GSK2245035, no treatment effect was observed in patients with mild asthma (weekly intranasal administration for 8 weeks), despite being well tolerated and capable of reducing nasal responsiveness upon allergen challenge in AR patients (Ellis, 2015; Ellis et al., 2017; Siddall et al., 2020).

In a pre-clinical murine model to investigate chronic allergic asthma induced by ragweed, the weekly intranasal application of the TLR9 agonist AZD1419 alleviated lung eosinophilia and concomitant IL-4, IL-5 and IL-13 levels in BALF (Campbell et al., 2014). Based on these findings, the efficacy of AZD1419 in the treatment of asthma was evaluated in a clinical trial. Patients received in total 13 weekly inhalations of AZD1419. But despite its safety and tolerability, the primary and secondary endpoints associated with the improvement of asthma symptoms reached no significance compared to placebo (Jackson et al., 2018; Psallidas et al., 2021). Still, the type 2-associated plasma cytokines TARC and monocyte-derived chemokine were lower than the baseline levels between weeks 4 and 28 of the study.

To modulate allergen-specific immune responses, TLR-ligand allergen fusion proteins combining allergens or allergen peptides with an inherent adjuvant activity were proposed and recently reviewed by Blanco-Pérez et al. (Blanco-Pérez et al., 2019). Despite promising preclinical data regarding the induction of immunotolerance by the fusion proteins, i.e., for the birch pollen allergen Bet v 1 fused to flagellin, or T cell peptides of the peach LTP Pru p 3 conjugated to TLR7 and TLR4 ligand, so far none of the candidates were clinically evaluated (Schülke et al., 2010; Losada Méndez et al., 2021; Goretzki et al., 2022).

As outlined above, the main idea of using TLR ligands is to modulate and amplify the adaptive immune response via induction of the innate immune system, however, the important question emerges to what extent does the repeated stimulation with TLR ligands rather induce the innate immune memory, leading to a reduced TLR responsiveness over time (Seeley and Ghosh, 2017). Based on this concept, prolonged and repeated administration of TLR ligands could result in innate immune ignorance, the opposite of the desired outcome. The repeated inhalation of the TLR9 agonist AZD1419, for instance, significantly reduced TLR9 RNA in peripheral blood of treated asthmatic patients (Psallidas et al., 2021).

Other novel treatment approaches are trying to directly antagonize or inhibit TLR-mediated responses. In a murine experimental set-up of AR induced by *D. pteronyssinus* extracts, airway hyperresponsiveness was abrogated in WT mice upon intrapulmonary administration of a TLR4 antagonist (TLR4-non-activating *Rhodobacter sphaeroides* LPS), and reduced lymphocytosis, eosinophilia, IL-5 and IL-13 levels in BALF (Hammad et al., 2009). Similar observations were made using an OVA model (adjuvant alum) and a short hairpin RNA to inhibit TLR4 (Xu et al., 2019). Although in humans, antagonizing TLRs in course of AIT has not been attempted yet, an IRAK4-degrading treatment (KT-474, ClinicalTrials.gov identifier: NCT04772885) is currently evaluated regarding its safety and tolerability in healthy volunteers and atopic dermatitis patients in a first-in-human clinical trial. IRAK4 is an important downstream protein kinase involved in TLR signal transduction. KT-474-induced IRAK4 knockdown in blood and skin lesions in atopic dermatitis patients was accompanied with the reduction of proinflammatory cytokines (Cooper, 2022).

Allergic diseases and TLR responses can also indirectly be modulated via probiotics shaping the gut microbiome. The composition of the gut microbiome is associated with the occurrence of allergic diseases. Recent meta-analyses provided evidence for a beneficial effect of probiotics in alleviating AR and atopic dermatitis symptoms (Luo et al., 2022; Umborowati et al., 2022), although clear associations between probiotic treatments shaping TLR responses in the context of allergic diseases are lacking. The gut microbiome is further influenced by the diet. In an allergic sensitization model, mice fed with an endotoxin and bacterial DNA-high diet had significantly lower Bet v 1-specific IgE and IgG1 levels compared to the endotoxin-low diet group, implying a role of activated TLRs in modulation of allergic responses (Schwarzer et al., 2017). Oral immunotherapy was shown to expand the biodiversity of the gut microbiome of peanut allergic patients (He et al., 2021). Human bronchial epithelial cells derived from patients undergoing HDM-sublingual AIT showed an increased responsiveness to a TLR3 ligand, however, the modulation of the airway microbiome was not evaluated within this study (Woehlk et al., 2023). The question remains if the influences of AIT on the microbiome are actually associated with the treatment effect or solely an unrelated consequence of the treatment.

## Conclusion

Taken together, the development of allergies is a multifactorial and highly complex process, underlying interconnected environmental and genetic factors that vary strongly among individuals. The evidence that TLRs contribute to the onset of allergic diseases is overwhelming, as demonstrated in several knockout models. As highlighted in Table 2, most allergen sources seem to interact with TLR4 in one way or another, with mainly LPS facilitating the adaptive immune response. Still, due to the complexity of the topic, many questions remain open: What is the contribution of the host´s microbiome? Studies on TLR activity influenced by alterations in the gut and mucosal microbiome between allergic and healthy individuals are scarce. To what extent are the microbiome and thereof derived ligands of an allergen source, such as pollen, actually influencing the host´s microbial diversity? Or is the pollen microbiome simply outcompeted by the mucosal microbiome? In this regard, it should be noted that *in vivo* studies investigating the contribution of TLRs in allergic sensitization are usually performed under pathogen-free conditions, which do not mimic the non-sterile environment in which humans naturally become sensitized. It is possible that TLRs might respond more sensitive under sterile conditions. Thus, human clinical trials addressing TLR pathways in the context of allergies are of particular interest and will certainly remain the focus of future studies.

## Figures and Tables

**Table:** 

**ACD**	allergic contact dermatitis
**AIT**	allergen-specific immunotherapy
**Alum**	aluminum hydroxide
**AR**	allergic rhinitis
**ATI**	alpha-amylase/trypsin inhibitors
**BALF**	bronchoalveolar lavage fluids
**BPE**	birch pollen extracts
**Breg**	B regulatory cells
**DAMP**	damage-associated molecular patterns
**DC**	dendritic cells
**GWAS**	genome-wide association studies
**HDM**	house dust mites
**HMGB1**	high mobility group box 1
**IFN**	interferon
**Ig**	immunoglobulin
**IL**	interleukin
**KO**	knock-out
**LMWHA**	low molecular weight hyaluronic acid
**LPS**	lipopolysaccharides
**MAPK**	mitogen-activated protein kinases
**MD-2**	myeloid differentiation factor 2
**moDC**	monocyte derived dendritic cells
**MPLA**	monophosphoryl lipid A
**MyD88**	myeloid differentiation primary response protein 88
**nsLTP**	non-specific lipid-transfer proteins
**OVA**	ovalbumin
**PAMP**	pathogen-associated molecular patterns
**PAO**	proteinase from *Aspergillus oryzae*
**PBMC**	peripheral blood mononuclear cell
**PDCD4**	programmed cell death 4
**poly (I:C)**	polyinosinic:polycytidylic acid
**PRR**	pattern recognition receptors
**RSV**	respiratory syncytial virus
**SNP**	single nucleotide polymorphism
**Th1/2/17**	T helper cell type 1/2/17
**TIR**	Toll/Interleukin-1 receptor
**TLR h/m**	Toll-like receptor human/murine
**TNF**	tumor necrosis factor
**TRIF**	TIR-domain-containing adapter-inducing interferon-β
**TSLP**	thymic stromal lymphopoietin
**WT**	wild-type
